# Associated factors of undernutrition in children with congenital heart disease: a cross-sectional study

**DOI:** 10.3389/fped.2024.1167460

**Published:** 2024-01-29

**Authors:** Xiaorui Ruan, Jun Ou, Yige Chen, Jingyi Diao, Peng Huang, Xinli Song, Jianhui Wei, Mengting Sun, Hongqiang Shi, Liuxuan Li, Jiapeng Tang, Hanjun Liu, Jiabi Qin

**Affiliations:** ^1^ Department of Epidemiology and Health Statistics, Xiangya School of Public Health, Central South University, Changsha, China; ^2^ Department of Cardiothoracic Surgery, Hunan Children's Hospital, Changsha, China; ^3^ Department of Clinical Pharmacology, Xiangya School of Pharmacy, Central South University, Changsha, China

**Keywords:** congenital heart disease, undernutrition, associated factors, dietary factor, gestational complication

## Abstract

**Objective:**

To evaluate the prevalence and associated factors of undernutrition among children with congenital heart disease (CHD) who have not undergone surgeries in China.

**Methods:**

This cross-sectional study included 734 CHD children along with their parents. The outcome of interest was undernutrition, including underweight, wasting, and stunting, defined as Z-scores (*i.e., weight-for-age, weight-for-height, and height-for-age*) ≤−2, according to the World Health Organization (WHO) growth standard. Exposures of interest, containing demographics, obstetric factors, maternal dietary factors, parents' life behaviors and habits, birth-related factors, cardiac-related factors, and preoperative factors, were analyzed using a multivariate logistic regression model to test their associations with undernutrition in CHD children.

**Results:**

Overall, 36.1%, 29.7%, and 21.3% of cases were underweight, wasted, and stunted, respectively. Multivariate logistic regression indicated that underweight was associated with demographic factors (including parents' occupational status, family income, and maternal body mass index pre-pregnancy), low birth weight (OR = 4.60, 2.76–7.70), pulmonary hypertension (OR = 4.46, 3.09–6.43), and pneumonia (OR = 1.88, 1.28–2.76). Artificially-fed children were 2.34 (1.36–4.01) times more likely to be underweight. Occupied mothers (OR = 0.62, 0.44–0.88) and fathers (OR = 0.49, 0.26–0.92) served as protective factors, while mothers having gestational complications (OR = 1.56, 1.11–2.18) and exposed to noisy environment (OR = 1.64, 1.11–2.42) during this pregnancy, and pulmonary hypertension (OR = 3.21, 2.30–4.49) increased the chance of wasting in offspring. The odds of being stunted were greater in families with >2 children (OR = 1.88, 1.13–3.14), placental abruption during this pregnancy (OR = 25.15, 2.55–247.89), preterm births (OR = 1.84, 1.02–3.31), low birth weight (OR = 3.78, 2.16–6.62), pulmonary hypertension (OR = 2.35, 1.56–3.53) and pneumonia (OR = 1.93, 1.28–2.90). In subgroup analyses, the associations differed between patients with different feeding patterns (breastfeeding *vs.* non-breastfeeding), CHD classifications (cyanotic vs*.* acyanotic), and prematurity (preterm vs*.* non-preterm).

**Conclusion:**

Undernutrition is common in preoperative CHD children. Familial demographics, maternal factors (including having gestational complications and exposure to noisy environment during pregnancy), and patient-related factors (encompassing preterm births, low birth weight, pulmonary hypertension, pneumonia, and feeding pattern) were found to contribute to undernutrition in CHD cases. However, associated factors among the three subgroups of distinct feeding patterns, CHD categorization, and prematurity exhibited varied outcomes, suggesting the necessity for targeted interventions.

## 1 Introduction

Across the globe, congenital heart disease (CHD) is the most prevalent birth defect and ranks the 7th leading cause of infant mortality (1), with the prevalence rising from 4.5 per 1,000 births in 1970–1974 to 9.4 per 1,000 births in 2010–2017 (2). Despite the fact that disability-adjusted life years (DALY) of CHD children aged under 10 years decreased by 41.6% from 1990 to 2019 (3), which is mainly attributed to the advancement and wide application of neonatal surgical interventions, as well as perioperative care, preoperative undernutrition is emerging as a global problem among CHD children, affecting their long-term outcomes.

According to World Health Organization (WHO), undernutrition refers to deficiencies in a child's intake of energy and/or nutrients, affecting 15%–64% of children with cardiac anomalies, and the proportion is higher in developing countries (4, 5). A case-control study in Nigeria indicated that preoperative CHD children were at higher risk of undernutrition than healthy counterparts (90.4% vs*.* 21.1%) (6). Based on our previous meta-analysis published in 2022, 27.4%, 24.4%, and 24.8% of preoperative CHD children globally suffered from underweight, wasting and stunting, respectively (7). Apart from general causes of undernutrition such as insufficient caloric intake and growing energy demand, several disease-related causes may be explanatory, including hypoxia (primary concern for cyanotic CHD), congestive heart failure (commonly seen in acyanotic CHD), pulmonary hypertension, and gastrointestinal dysfunction as a result of complications (5, 8, 9). A number of articles have sufficiently narrated that undernutrition in CHD children is associated with worse hospital outcomes, higher infection rates, increased morbidity, mortality and complications (10, 11), impaired neurodevelopment, and poor social and academic performance (12, 13), which exert long-term negative impact on the overall development until adulthood.

Previous investigators have demonstrated associated factors of undernourishment among CHD children who had not performed cardiac surgeries, ranging from patients' demographics (including age, gender, residence, family size and income, etc.), to their cardiac factors (containing cyanotic heart disease, pulmonary hypertension, heart failure, anemia, etc.) (14–16). Maternal-related factors, which are comprised of elder age, low education level, unemployment, high anxiety and depression level, were also reported to be associated with offspring's growth retardation (17, 18). In addition, a few studies explored father's demographic characteristics as associated factors (15, 16, 19). Noticeably, Vaidyanathan et al. performed 24-h dietary recall on mothers and found that fat intake below 50 g per day was associated with underweight and stunting in CHD children (16), implying that maternal unbalanced dietary pattern adversely impacts nutritional status in the offspring. However, there is a lack of research documenting the relationship between maternal periconceptional dietary factors and their CHD offspring's undernourishment, as well as obstetric factors (i.e., history of pregnancy outcomes and gestational complications during this pregnancy), life behaviors and habits and exposure to environmental hazards during pregnancy. Considering that only 38.7% of syndromic CHD infants were breastfed reaching 6 months mainly due to sucking inactivity (20), probing predictors of undernutrition independently among breastfed and non-breastfed cases were of vital significance. Furthermore, preceding publications reached heterogenous results, particularly on influencing factors of undernutrition in cyanotic and acyanotic cases (15, 21). Attention should also be paid to nutritional status of preterm CHD births, defined as born <37 weeks of gestation, as they exhibit different growth patterns with full-term infants (22). Therefore, we conducted a cross-sectional study to investigate the associated factors of underweight, wasting and stunting among preoperative CHD children in China, and analyzed these factors separately based on feeding patterns, cyanosis, and prematurity, so as to lay foundation for targeted interventions to mitigate preoperative undernutrition.

## 2 Materials and methods

### 2.1 Ethics compliance

This study was approved by the Ethics Committee of Xiangya School of Public Health, Central South University. We have obtained written informed consent from all participants, and this research has been registered in the Chinese Clinical Trial Registration Center (Registration Number: ChiCTR1800016635).

### 2.2 Study population

This was a cross-sectional study conducted in the Department of Cardiothoracic Surgery of Hunan Children's Hospital between November 2017 and January 2021, which was part of a larger case-control study, thus the procedure and related details have been previously described (23).

All non-syndromic CHD children aged 0–5 years, of Han Chinese descent, who had not undergone cardiac surgery, and their parents were enrolled in this study. These patients had been diagnosed with CHD after screening for symptoms, physical examination, and echocardiography, according to the 10th version of the International Classification of Diseases (ICD-10). The exclusion criteria were: (1) cases who underwent emergency surgeries; or (2) with severely impaired liver and kidney function; or (3) diabetics; or (4) patients and/or his or her parents who lack relevant information. Worthy of mentioning, children who have congenital metabolic disorders, lesions in other organ systems apart from the cardiovascular system, genetic syndromes or chromosomal aberrations (e.g., Down's syndrome, catarrhal syndrome, Noonan syndrome, Holt–Oram syndrome, etc.) were not taken into consideration.

### 2.3 Sample size calculation

The sample size calculation was based on the prevalence rates of underweight (26.2%), wasted (24.6%), and stunted (22.3%) CHD children observed in a previous study (24). Additionally, we used the formula for calculating sample size in a cross-sectional study. Assuming a margin of error (*d*) of 0.15*p (*p* stands for prevalence), we calculated the required sample sizes for underweight, wasted, and stunted children as 481, 524, and 595, respectively. To ensure adequate statistical power, we selected the largest sample size of 595 as the final sample size for our study. After considering a non-response rate of 5%, the ultimate sample size was determined to be 625.

$$n=\frac{Z_{1-\alpha /2}^{2}*p(1-p)}{d^{2}}$$

### 2.4 Exposures of interest

At the time of hospital admission, eligible parents were asked to complete a self-designed questionnaire during face-to-face interviews conducted by health professionals trained in the survey and supervised by the principal investigator to minimize investigation bias. The questionnaire contained items on parents' demographic characteristics, life behaviors and habits, maternal history of adverse pregnancy outcomes, family history of congenital malformation, gestational complications during this pregnancy, periconceptional dietary factors, intake of folic acid, and history of exposure to environmental hazards, along with CHD children's demographic characteristics, birth-related factors, cardiac-related factors, preoperative factors, and feeding pattern. Additionally, CHD subtypes determined by echocardiography results, diagnosis of cardiac complications were retrieved from medical records.

The following maternal features were collected, including (1) demographic characteristics (*i.e., age at this pregnancy, residence, education level, occupational status, family annual income, number of children, and body mass index before this pregnancy*), (2) history of adverse pregnancy outcomes which is the sum of history of adverse birth outcomes (*i.e., history of spontaneous abortion, history of stillborn fetus, history of premature birth, history of low birth weight, and history of intrauterine growth arrest*) and history of gestational complications (*i.e., history of gestational diabetes mellitus, history of gestational hypertension, history of placenta previa, history of premature rupture of membrane, history of antepartum/postpartum hemorrhage, history of anemia during pregnancy, and history of ectopic gestation*), (3) family history of congenital malformation, (4) gestational complications during this pregnancy (*i.e., gestational diabetes mellitus, gestational hypertension, placenta previa, placental abruption, premature rupture of membrane, antepartum/postpartum hemorrhage, and anemia during pregnancy*), (5) periconceptional dietary factors (*i.e., eating pickles, eating preserved eggs, eating salted eggs, eating smoked food, eating fried food, eating dairy products, eating eggs, eating vegetables, eating fruits, eating beans, eating meat, and eating seafood*), (6) life behaviors and habits before or during early stage of pregnancy (*i.e., smoking, passive smoking, drinking, drinking tea, drinking coffee, using computers, using cellphones, and experiencing negative events*), (7) intake of folic acid, and (8) history of exposure to environmental hazards (*i.e., exposure to air pollution, exposure to a noisy environment, exposure to newly renovated houses, and exposure to radiation*).

In addition, the father's demographic characteristics (*i.e., education level and occupational status*) and life behaviors (*i.e., smoking, drinking, drinking tea, drinking coffee, using computers, and using cellphones*) were considered as well.

For CHD children, we collected demographic characteristics (*i.e., age, gender*), birth-related factors (*i.e., mode of delivery, premature birth, low birth weight, and multiple births*), CHD subtypes and complications (*i.e., cyanosis, hemodynamics, pulmonary hypertension, pneumonia*), and feeding pattern. Detailed definitions of exposures of interest listed above are shown in Supplementary Table S1.

Furthermore, a perinatal health care handbook (PHCH) is distributed to each Chinese pregnant woman, recording basic demographic characteristics, lifestyle behaviors, family history of congenital malformations (patients' first and second degree relatives), history of various diseases (including history of previous and current gestational complications), diverse medical examinations during pregnancy, and folic acid supplementation. Relevant information from PHCH and medical records were extracted to confirm the corresponding results, which dramatically reduced recall bias.

### 2.5 Outcomes of interest

The outcome of interest was undernutrition, classified into underweight, wasting, and stunting. At the time of hospital admission, measurements of weight and height were carried out on each patient. Anthropometric measurements and subsequent calculation of three indicators were performed according to 2006 WHO child growth standards (available at: https://www.who.int/toolkits/child-growth-standards).

After zeroing the weight scale, children were weighed 2 h after eating or in fasted state, with clothes and socks removed until minimal clothes. Weight readings were recorded after stabilization, to the nearest 0.1 kg. Upon weighing, infants and children within 2 years old had their lengths measured in the supine position, the body kept straight, with the top of head and soles of feet touching two edges of the measuring board. Heights of children over 2 years old were measured when standing, eyes straight ahead, chest slightly upright, abdomen slightly retracted, arms naturally hanging down, heels closing together, toes 60 degrees apart, scapulae, hips and heels touching the column of the stadiometer (sensitivity of 0.1 cm).

Weight for age *Z*-score (WAZ), weight for height *Z*-score (WHZ), and height for age *Z*-score (HAZ), were then calculated via WHO Anthro software (access at: http://www.who.int/childgrowth/software/en/), based on the following formula.

$$Z\;-\;\mathrm{score}=\frac{(\mathrm{observed}\;\mathrm{value})-(\mathrm{median}\;\mathrm{reference}\;\mathrm{value})}{z\;-\;\mathrm{score}\;\mathrm{of}\;\mathrm{the}\;\mathrm{reference}\;\mathrm{population}}$$

Underweight, wasting, and stunting were defined if WAZ ≤−2, WHZ ≤−2, HAZ ≤−2, respectively. Of note, to facilitate comparison with other publications, we merged severe undernutrition (*i.e.*, severe underweight, severe wasting, and severe stunting), which was defined as *Z*-scores ≤−3, into undernutrition.

### 2.6 Statistical analysis

Non-normally distributed values including age were presented as medians and interquartile range (IQR), and categorical variables containing exposures and outcomes were expressed as absolute and percentage distribution. Missing values were interpolated using means or medians. Chi-squared tests or Fisher's exact tests were used to examine the differences of unordered categorial variables between two groups. For variates of significance, Odds ratios (ORs) and 95% confidence intervals (CIs), along with P-values were later computed by multivariable logistic regression analysis. Statistical analysis was performed using SPSS software. Two-sided *P* < 0.05 was considered statistically significant.

### 2.7 Subgroup analyses

Subgroup analyses were performed between breastfed and non-breastfed children, cyanotic and acyanotic children, and children born with or without prematurity. To facilitate calculation, artificially feeding and mixed feeding children were combined into non-breastfed group. Comprehensive definitions were provided in Supplementary Table S1. The statistical analysis followed the same protocol as primary analysis, as described in Section 2.6.

## 3 Results

### 3.1 Prevalence of underweight, wasting, and stunting

From November 2017 to January 2021, 753 CHD children meeting inclusion criteria, along with their parents, were recruited for this study. 19 of them met exclusion criteria (10 cases had genetic syndromes or chromosomal aberrations, 4 cases had liver or kidney failure, 5 cases had single parent and lack relevant information) and were excluded. Thus, 734 children and their parents were eligible for following analysis. Over half of the cases (55.3%) were male, and the median age was 9 months (IQR: 4–24). 40.2% of cases (*n* = 295) were admitted to the hospital before 6 months of age, and the distribution of age at admission was shown in Supplementary Figure S1. The majority of children lived in rural areas (65.5%), with more than two other family members (84.6%), who altogether earned <50,000 RMB annually (54.6%). Of the total, 12.9% (*n* = 95) participants were born prematurely and 13.4% (*n* = 98) individuals were of low birth weight. There was a similar proportion of children born by spontaneous vaginal delivery (*n* = 383, 52.2%) and caesarean section (*n* = 351, 47.8%). The most common malformations among CHD patients at admission were: ventricular septal defect (69.3%), patent foramen oval (62.4%) patent ductus arteriosus (44.1%), and atrial septal defect (23.8%). These CHD subtypes were mostly acyanotic heart disease (88.1%). Pulmonary hypertension and pneumonia were documented in 39.6% and 31.6% of the patients, respectively.

The overall prevalence of undernutrition was 48.1% (*n* = 353, 95% CI: 44.5%–51.7%) of the 734 CHD children, and 6.4% (*n* = 47) of whom showed all the abnormal parameters, containing WAZ, WHZ, HAZ <−2 simultaneously. The prevalence of children underweight, wasted, and stunted was 36.1% (*n* = 265, 95% CI: 32.6%–39.6%), 29.7% (*n* = 218, 26.4%–33.0%), and 21.3% (*n* = 158, 18.3%–24.2%), respectively. Baseline characteristics are shown in Table 1.

**Table 1:** Baseline characteristics of patients with CHD.

Variables	Cases (percentage, %)	Variables	Cases (percentage, %)
Gender		Classification of heart disease	
Male	406 (55.3)	Acyanotic	647 (88.1)
Female	328 (44.7)	Cyanotic	87 (11.9)
Residence location		Pulmonary hypertension	
Rural area	481 (65.5)	No	443 (60.4)
Urban area	253 (34.5)	Yes	291 (39.6)
Family annual income (RMB)		Pneumonia	
<50,000	401 (54.6)	No	502 (68.4)
50,000–100,000	240 (32.7)	Yes	232 (31.6)
≥100,000	93 (12.7)	Underweight	
Number of family members		No	469 (63.9)
≤3	113 (15.4)	Yes	265 (36.1)
>3	621 (84.6)	Wasting	
Premature		No	516 (70.3)
No	639 (87.1)	Yes	218 (29.7)
Yes	95 (12.9)	Stunting	
Low birth weight		No	578 (78.7)
No	636 (86.6)	Yes	156 (21.3)
Yes	98 (13.4)		

### 3.2 Univariate analysis of associated factors of undernutrition in CHD children

Results of the association between undernutrition and maternal factors, paternal factors, and CHD children's factors were displayed in Supplementary Tables S2–S5.

Being underweight was significantly associated with maternal occupational status (unemployed), family annual income (<50,000 RMB), number of children (>2), body mass index before this pregnancy (underweight), history of intrauterine growth arrest, gestational complications during this pregnancy, premature rupture of the membrane during this pregnancy, never eating seafood. Additionally, the following influence factors were also considered explanatory, including paternal educational level (high school and below) and occupational status (unemployed), born prematurely, of low birth weight, multiple births, cyanotic heart disease, pulmonary hypertension, pneumonia and feeding pattern (artificial feeding).

About wasting in CHD children, parents’ occupational status (unemployed), paternal education level (high school and below), family annual income (<50,000 RMB), history of intrauterine growth arrest, gestational complications during this pregnancy, maternal exposure to a noisy environment were significant predictors. On top of that, pulmonary hypertension, pneumonia and feeding pattern (artificial feeding) emerged as significant risk factors.

With respect to stunted CHD children, the significant associate factors included maternal number of children (>2), gestational hypertension during this pregnancy, placental abruption during this pregnancy, and premature rupture of membrane during this pregnancy. Moreover, mode of delivery (caesarean section), born prematurely, of low birth weight, multiple births, pulmonary hypertension, pneumonia, and feeding pattern (artificial feeding) were significantly associated with stunting.

### 3.3 Multivariable logistic regression analysis for associated factors of undernutrition in CHD children

Multivariate logistic regression analysis was performed by forward stepwise procedure, using significant variables from univariate analysis, and the results of underweight, wasting and stunting are shown in Tables 2–4 respectively.

**Table 2:** Associated factors of underweight in CHD children based on multiple logistic regression analysis.

Variables	*β*	SE	Wald*χ*^2^	OR (95% CI)	*P*
Mother's occupational status	−0.456	0.181	6.354	0.63 (0.44–0.90)	0.012
Father's occupational status	−0.807	0.360	5.023	0.45 (0.22–0.90)	0.025
Family annual income (RMB)					
50,000–100,000	−0.684	0.203	11.363	0.50 (0.34–0.75)	0.001
≥100,000	−0.307	0.285	1.157	0.74 (0.42–1.29)	0.282
Body mass index before this pregnancy					
18.5–23.9 (healthy)	−0.697	0.242	8.322	0.50 (0.31–0.80)	0.004
≥24.0 (overweight/obese)	−0.871	0.299	8.457	0.42 (0.23–0.75)	0.004
Low birth weight	1.527	0.262	34.031	4.60 (2.76–7.69)	<0.001
Pulmonary hypertension	1.495	0.187	64.085	4.46 (3.09–6.43)	<0.001
Pneumonia	0.631	0.196	10.322	1.88 (1.28–2.76)	0.001
Feeding pattern					
Artificial feeding	0.849	0.276	9.489	2.34 (1.36–4.01)	0.002
Mixed feeding	0.084	0.196	0.184	1.09 (0.74–1.60)	0.668

**Table 3:** Associated factors of wasting in CHD children based on multiple logistic regression analysis.

Variables	*β*	SE	Wald*χ*^2^	OR (95% CI)	*P*
Mother's occupational status	−0.472	0.173	7.420	0.62 (0.44–0.88)	0.006
Father's occupational status	−0.714	0.324	4.856	0.49 (0.26–0.92)	0.028
Gestational complications during this pregnancy	0.442	0.171	7.420	1.56 (1.11–2.18)	0.010
Exposure to noisy environment	0.493	0.200	6.075	1.64 (1.11–2.42)	0.014
Pulmonary hypertension	1.167	0.171	46.636	3.21 (2.30–4.49)	<0.001

**Table 4:** Associated factors of stunting in CHD children based on multiple logistic regression analysis.

Variables	*β*	SE	Wald*χ*^2^	OR (95% CI)	*P*
Number of children	0.633	0.262	5.851	1.88 (1.13–3.14)	0.016
Placental abruption during this pregnancy	3.225	1.167	7.632	25.15 (2.55–247.89)	0.006
Premature birth	0.609	0.300	4.130	1.84 (1.02–3.31)	0.042
Low birth weight	1.329	0.286	21.560	3.78 (2.16–6.62)	<0.001
Pulmonary hypertension	0.853	0.208	16.859	2.35 (1.56–3.53)	<0.001
Pneumonia	0.655	0.208	9.920	1.93 (1.28–2.90)	0.002

CHD children, raised by working mother (OR = 0.63, 95% CI: 0.44–0.90) or father (OR = 0.45, 0.22–0.90), had lower odds of underweight than those nurtured by jobless parents. Compared to CHD children whose family annual income was lower than 50,000 RMB, those with household income ranging 50,000–100,000 RMB per year were 50.4% (33.9%–75.1%) times less likely to be underweight. Normal body mass index and being overweight or obese before this pregnancy emerged as protective factors, as the chance of underweight decreased by 50.2% (20.0%–69.0%) and 58.1% (24.7%–76.7%), respectively. On the other hand, the possibility of being underweight increased 4.60 (2.76–7.69) times, 4.46 (3.09–6.43) times, 1.88 (1.28–2.76) times, 2.34 (1.36–4.01) times in CHD children of low birth weight, with pulmonary hypertension, with pneumonia, fed artificially, respectively, as is described in Table 2.

Maternal history of gestational complications during this pregnancy, and their exposure to a noisy environment raised by 55.6% (11.2%–117.7%), 63.7% (10.6%–42.3%) the chance of wasting in CHD children, respectively. Patients with pulmonary hypertension were 3.21 (2.30–4.49) times more likely to be wasted. Nevertheless, mothers and fathers who were office workers reduced the risk of wasting by 37.6% (12.4%–55.6%), 51.0% (7.6%–74.0%), respectively, based on Table 3.

The number of children was positively associated with stunting in CHD children, as those who had siblings were 1.88 (1.13–3.14) times more likely to be stunted. The odds of stunting dramatically rose when mothers had placental abruption during this pregnancy (OR = 25.15, 2.55–247.89). Additionally, cases who were born prematurely, were of low birth weight, had pulmonary hypertension and pneumonia were 1.84 (1.02–3.31) times, 3.78 (2.16–6.62) times, 2.35 (1.56–3.53) times, 1.93 (1.28–2.90) times more risky to be stunted, respectively. Importantly, placental abruption during this pregnancy had the strongest association. Table 4 showed the results.

### 3.4 Subgroup analyses

Subgroup analyses were conducted on breastfed and non-breastfed children, cyanotic and acyanotic children, preterm and non-preterm children, to assess whether feeding pattern, disease severity, and prematurity play a role. The results were shown in Tables 5–7, respectively.

**Table 5:** Associated factors of underweight, wasting, and stunting in breastfed and non-breastfed CHD children based on multiple logistic regression analysis.

Growth indicator	Variables	*β*	SE	Wald*χ*^2^	OR (95% CI)	*P*
Underweight	Breastfed					
	Low birth weight	1.600	0.482	11.008	4.95 (1.92–12.74)	0.001
	Pulmonary hypertension	1.801	0.277	42.283	6.06 (3.52–10.42)	<0.001
	Eating seafood					
	Sometimes	−0.231	0.402	0.331	0.79 (0.36–1.75)	0.565
	Always	−0.977	0.447	4.771	0.38 (0.16–0.91)	0.029
	Non-breastfed					
	Annual family income (RMB)					
	50,000–100,000	−0.827	0.263	9.859	0.44 (0.26–0.73)	0.002
	≥100,000	−0.180	0.369	0.238	0.84 (0.41–1.72)	0.625
	Body mass index before this pregnancy					
	18.5–23.9 (healthy)	−0.905	0.318	8.128	0.40 (0.22–0.75)	0.004
	≥24.0 (overweight/obese)	−1.42	0.398	12.752	0.24 (0.11–0.53)	<0.001
	Father's occupational status	−0.976	0.435	5.042	0.38 (0.16–0.88)	0.025
	Low birth weight	1.381	0.303	20.752	3.98 (2.20–7.21)	<0.001
	Pulmonary hypertension	1.242	0.243	26.242	3.46 (2.15–5.57)	<0.001
	Pneumonia	0.808	0.248	10.605	2.24 (1.38–3.65)	0.001
Wasting	Breastfed					
	Pulmonary hypertension	1.462	0.264	30.749	4.31 (2.57–7.23)	<0.001
	Non-breastfed					
	Mother's occupational status	−0.566	0.231	5.992	0.57 (0.36–0.89)	0.014
	Body mass index before this pregnancy					
	18.5–23.9 (healthy)	−0.671	0.294	5.206	0.51 (0.29–0.91)	0.023
	≥24.0 (overweight/obese)	−1.222	0.394	9.616	0.30 (0.14–0.64)	0.002
	Pulmonary hypertension	1.087	0.232	7.297	2.97 (1.88–4.67)	<0.001
	Gestational complications during this pregnancy	0.626	0.232	7.297	1.87 (1.19–2.94)	0.007
Stunting	Breastfed					
	Residence	−1.083	0.467	5.377	0.34 (0.14–0.85)	0.020
	Mother's education level					
	High school	−0.344	0.386	0.794	0.71 (0.33–1.51)	0.373
	College and above	−1.434	0.566	6.406	0.24 (0.08–0.72)	0.011
	Low birth weight	2.307	0.487	22.429	10.04 (3.87–26.08)	<0.001
	Pulmonary hypertension	0.806	0.341	5.597	2.24 (1.15–4.36)	0.018
	Non-breastfed					
	Low birth weight	1.373	0.291	22.23	3.95 (2.23–6.98)	<0.001
	Pulmonary hypertension	0.735	0.257	8.20	2.09 (1.26–3.45)	0.004
	Pneumonia	0.833	0.256	10.61	2.30 (1.39–3.80)	0.001
	Paternal drinking	0.529	0.244	4.71	1.70 (1.05–2.74)	0.030

**Table 6:** Associated factors of underweight, wasting, and stunting in cyanotic and acyanotic CHD children based on multiple logistic regression analysis.

Growth indicator	Variables	*β*	SE	Wald*χ*^2^	OR (95% CI)	*P*
Underweight	Acyanotic CHD					
	Mother's occupational status	0.414	0.201	4.241	0.66 (0.45–0.98)	0.039
	Family annual income (RMB)					
	50,000–100,000	0.562	0.229	6.046	0.57 (0.36–0.89)	0.014
	≥100,000	0.127	0.310	0.169	0.88 (0.48–1.62)	0.681
	Body mass index before this pregnancy					
	18.5–23.9 (healthy)	0.752	0.258	8.503	0.47 (0.28–0.78)	0.004
	≥24.0 (overweight/obese)	0.835	0.320	6.818	0.43 (0.23–0.81)	0.009
	Family history of congenital malformation	0.894	0.382	5.489	2.44 (1.16–5.16)	0.019
	Low birth weight	1.345	0.324	17.244	3.84 (2.03–7.25)	<0.001
	Pulmonary hypertension	1.563	0.205	57.924	4.77 (3.19–7.14)	<0.001
	Pneumonia	0.499	0.212	5.531	1.65 (1.09–2.50)	0.019
	Feeding pattern					
	Artificial feeding	0.881	0.306	8.320	2.41 (1.33–4.39)	0.004
	Mixed feeding	0.209	0.212	0.972	1.23 (0.81–1.87)	0.324
	Cyanotic CHD					
	Pulmonary hypertension	1.192	0.524	5.185	3.29 (1.18–9.19)	0.023
Wasting	Acyanotic CHD					
	Mother's occupational status	0.494	0.192	6.646	0.61 (0.42–0.89)	0.010
	Body mass index before this pregnancy					
	18.5–23.9 (healthy)	0.663	0.244	7.405	0.52 (0.32–0.83)	0.007
	≥24.0 (overweight/obese)	0.901	0.309	8.509	0.41 (0.22–0.74)	0.004
	Family history of congenital malformation	0.721	0.359	4.038	2.06 (1.02–4.15)	0.044
	Exposure to noisy environment	0.563	0.217	6.716	1.76 (1.15–2.69)	0.010
	Pulmonary hypertension	1.191	0.196	36.809	3.29 (2.24–4.83)	<0.001
	Cyanotic CHD					
	Paternal education level	2.788	1.199	5.406	0.06 (0.01–0.65)	0.020
	History of gestational hypertension	2.824	1.366	4.275	16.84 (1.16–244.85)	0.039
Stunting	Acyanotic CHD					
	Number of children	0.609	0.293	4.315	1.84 (1.04–3.27)	0.038
	Low birth weight	1.205	0.325	13.747	3.34 (1.76–6.31)	<0.001
	Pulmonary hypertension	0.833	0.224	13.803	2.30 (1.48–3.57)	<0.001
	Pneumonia	0.558	0.230	5.899	1.75 (1.11–2.74)	0.015
	Feeding pattern					
	Artificial feeding	0.789	0.326	5.870	2.20 (1.16–4.17)	0.015
	Mixed feeding	0.384	0.239	2.580	1.47 (0.92–2.35)	0.108
	Cyanotic CHD					
	Low birth weight	1.558	0.691	5.091	4.75 (1.23–18.38)	0.024

**Table 7:** Associated factors of underweight, wasting, and stunting in preterm and non-preterm CHD children based on multiple logistic regression analysis.

Growth indicator	Variables	*β*	SE	Wald*χ*^2^	OR (95% CI)	*P*
Underweight	Non-preterm CHD children					
	Paternal occupational status	−1.116	0.383	8.505	0.33 (0.15–0.69)	0.004
	Low birth weight	1.524	0.386	15.565	4.59 (2.15–9.79)	<0.001
	Pulmonary hypertension	1.421	0.201	50.178	4.1 4 (2.80–6.14)	<0.001
	Pneumonia	0.526	0.210	6.274	1.69 (1.12–2.55)	0.012
	Feeding pattern					
	Artificial feeding	0.989	0.308	10.285	2.69 (1.47–4.92)	0.001
	Mixed feeding	0.047	0.209	0.050	1.05 (0.70–1.58)	0.824
	Preterm CHD children					
	Low birth weight	1.182	0.492	5.773	3.26 (1.24–8.55)	0.016
	Pulmonary hypertension	1.627	0.527	9.514	5.09 (1.81–14.31)	0.002
Wasting	Non-preterm CHD children					
	Maternal occupational status	−0.470	0.192	6.032	0.62 (0.43–0.91)	0.014
	Paternal occupational status	−1.045	0.371	7.934	0.35 (0.17–0.73)	0.005
	Pulmonary hypertension	1.461	0.194	56.599	4.31 (2.95–6.31)	<0.001
	Pneumonia	0.403	0.200	4.035	1.50 (1.01–2.22)	0.045
	Preterm CHD children					
	No significant associated factors					
Stunting	Non-preterm CHD children					
	Placental abruption during this pregnancy	3.376	1.227	7.571	29.24 (2.64–323.73)	0.006
	Low birth weight	1.497	0.354	17.831	4.47 (2.23–8.95)	<0.001
	Pulmonary hypertension	0.765	0.236	10.512	2.15 (1.35–3.41)	0.001
	Pneumonia	0.594	0.241	6.079	1.81 (1.13–2.90)	0.014
	Preterm CHD children					
	Maternal occupational status	−1.344	0.494	7.390	0.26 (0.10–0.69)	0.007
	Pulmonary hypertension	1.215	0.503	5.836	3.37 (1.26–9.04)	0.016

Pulmonary hypertension was the mutual predictive factor of undernutrition in both breastfed and non-breastfed children. Children of low birth weight were prone to be underweight and stunted, regardless of their feeding patterns. It is worth mentioning that mothers eating seafood ≥3 times per week dramatically decreased the chance of being underweight in breastfed children (OR = 0.38, 0.16–0.91). Mothers who were occupied and whose body mass index was ≥18.5 lowered the possibility of being wasted for their non-breastfed children, while having gestational complications during this pregnancy increased the hazard. Living in urban areas, mothers receiving higher education served as protective factors for stunting in breastfed children. Non-breastfed children who were diagnosed with pneumonia and whose fathers drank alcohols had higher odds of being stunted.

In acyanotic children, whose mothers unoccupied, with body mass index less than 18.5 before this pregnancy, with family history of congenital malformation were the risk factors for being underweight and wasted. Artificial and mixed feeding adversely impacted children's growth, and elevated the rate of underweight and stunting. However, for children who presented cyanosis, we only found associations between pulmonary hypertension and underweight (OR = 3.29, 1.18–9.19), paternal education level (OR = 0.06, 0.01–0.65), history of gestational hypertension (OR = 16.84, 1.16–244.85) and wasting, low birth weight and stunting (OR = 4.75, 1.23–18.38).

In both preterm and non-preterm children, the presence of pulmonary hypertension increased the likelihood of being underweight and stunted. Additionally, pneumonia was found to be correlated with undernutrition in full-term children (OR = 1.69, 1.12–2.55), but this association was not observed among preterm births. Importantly, low birth weight was found to be associated with both preterm (OR =  3.26, 1.24–8.55) and non-preterm children (OR = 4.59, 2.15–9.79), suggesting a potential interaction between prematurity and low birth weight.

## 4 Discussion

The study was designed to extend our understanding of the relationship between preoperative undernutrition in CHD patients and associated factors in China context. We provided new evidence that gestational complications during this history, maternal exposure to a noisy environment, and artificial feeding increased the odds of undernutrition in their offspring. Additionally, our subgroup analyses have revealed the necessity of implementing distinct interventions tailored to CHD children across diverse categories, such as prematurity, breastfeeding status, as well as distinguishing between those with cyanotic CHD and acyanotic CHD.

In our study, the overall prevalence of underweight, wasting and stunting among CHD children preoperatively reached 36.1%, 29.7%, and 21.3%, respectively. Similar results were shown in previous studies among Chinese and Thai CHD children (15, 17, 25). Qin et al. reported that 25.3%, 25.3%, and 28.6% of Chinese CHD cases were underweight, wasted, and stunted, respectively (17). In this study, children aged 2–4 years with simple repair (SR) CHD, and their mothers were included, while preterm were excluded. We observed a higher proportion of underweight in CHD children because patients with severe CHD subtypes, preterm, and infants who were vulnerable to undernutrition were included in our study. In contrast, parameters of insufficient growth documented in other developing countries were higher than our results (14, 19, 21, 26). For instance, Batte et al. illustrated that, in Uganda, the proportion of underweight, wasting, and stunting in children aged under 5 years was 44.8%, 31.5%, and 48.3%, respectively (14). Studies in Nigeria (21), Egypt (26), and Ethiopia (27), showed 42.9%, 61.9%, and 49.1% of the subjects suffered underweight, wasted and stunted, respectively. Higher rates may be explained by differences in the economy level, availability of medical resources, and inclusion criteria of participants. Differ from our observation, lower prevalence was documented by the research in France, as underweight and wasted children accounted for 14% and 17% of CHD infants (28), which may be attributed to different standards for outcomes.

Contradicting to the former study that working mothers tend to have children with impaired linear growth due to inadequate childcare time (29), we found that employed parents were more likely to raise healthy CHD children. A probable explanation is that employed parents, with stable income and greater financial resources, are more likely to possess a heightened understanding of CHD, ensuring that their children receive timely medical treatment and maintain proper attention to nutritional status. Compared to the only child, a higher proportion of children with siblings were observed to be stunted, supported by Vaidyanathan et al. (16), indicating that caregivers ought to pay more attention to the children's feeding and chronic nutritional status, especially when they have more than one child to attend. Cases, with maternal BMI <18.5 before pregnancy were more likely to be underweight. Likewise, a cohort study in Japan found maternal pre-conception underweight increased the odds of adverse outcomes in neonates, including preterm birth, low birth weight, and small-for-gestational-age (30). Our study furthered evidence about potential long-term effect of maternal low BMI on growth inadequacy among CHD children.

Obstetric factors were evaluated to be potential predictors of growth retardation. A handful of studies have articulated that adverse pregnancy outcomes elevated the odds of perinatal death and morbidity, particularly growth restriction (31–33). Downes et al. performed a systematic review, finding the association between placental abruption and low birth weight, intrauterine growth restriction, and mortality (31). A Danish cohort study proved that premature rupture of membrane mediated spontaneous preterm birth in CHD neonates (32). However, it remains unclear about the relationship between adverse pregnancy outcomes and undernourishment in children with cardiac malformations. We initially reported relevant evidence showing a significant correlation between chronic undernutrition, indicated by stunting, and placental abruption during pregnancy, using a multivariate logistic regression model. Additionally, newborns were more likely to suffer wasting if theirs had history of gestational complications during this conception. Univariate analysis showed that gestational hypertension and premature rupture of membrane during this gestation played a part in faltering growth. Dysregulation of utero circulation and inadequate vessel supply, resulting in fetal hypoxia, acidosis, and subsequent immature growth of gastrointestinal tract, might be explanatory for malabsorption and delayed growth. It is interesting to note the relationship between history of intrauterine growth arrest before this pregnancy and undernutrition in the offspring. Nevertheless, there is lack of literature on the underlying mechanisms, highlighting the need for future research in this area.

To our knowledge, this is the first study represents the first attempt to the possible association of maternal dietary factors, life behaviors and habits, exposure to environmental hazards exert effect, and nutritional status of preoperative CHD children. We found children's nutrition was positively associated with maternal consumption of healthy food, containing eggs, vegetables, fruits, beans, but the association did not reach statistical significance. Children, whose mothers never eat seafood (e.g., fish), defined as less than once per week, had higher odds of being underweight, while mothers eating seafood ≥3 times per week served as a protective factor of underweight in breastfed children. Generally shaped by sociocultural settings, family dietary patterns, particularly maternal feeding behaviors, influence children's food preferences and overall growth (34). In terms of parent's life behaviors and habits during periconceptional period, our former study demonstrated that maternal active and passive smoking, whether before pregnancy or during early phase of pregnancy, were risk factors for CHD in offspring (35). Whereas, no significant association between parental smoking, along with other behaviors and undernutrition in children was observed in our study. Importantly, we acquired that a greater proportion of subjects experienced wasting with mothers exposed to a noisy environment during gestation. As also evidenced by a British study that exposure-response relationship existed between traffic noise and low birth weight, suggesting that noise-induced activation of sympathetic nervous system and successive reduction in blood flow through uterus posed threat to infants (36, 37), and may exert lasting influence on children's nourishment post birth.

It was observed that low birth weight was the risk factor for underweight and stunting, in line with earlier studies among CHD subjects (16, 28, 38). Our subgroup analysis on children born with or without prematurity suggested a potential interaction between preterm birth and low birth weight in relation to children's undernutrition. Interestingly, newborns delivered via caesarean section had an increased susceptibility to be stunted, although our analysis did not reveal a statistically significant association in multivariate logistic model. This mode of delivery disturbs colonization of microbes which are naturally transmitted from mothers through vaginal delivery, thereby this altered composition of gut microbiota might enhance the chance of opportunistic infection and diminish absorption of nutrients, leading to long-term damage to somatic development (39, 40).

Furthermore, we found that children with cyanotic heart disease bore a greater risk of growth deficiency, yet the association was not proved to be statistically significant in multivariate logistic regression, which was in accordance with current studies (14, 41). On the contrary, a Chinese study among postoperative children reported that acyanotic CHD patients tended to be underweight and cyanotic ones were prone to be stunted (20). Opposing this finding, Hassan et al. stated that acyanotic heart disease was linked with stunting in Egyptian CHD children (26). With regard to the discrepancy among published papers, diverse study populations (have or have not undergone surgeries) and classifications of cyanotic heart disease cannot be ruled out. Convincing systematic review and meta-analysis concerning differences in the correlation did not occur to date. In our subgroup analysis, huge heterogeneity of factors associated with undernutrition was observed between cyanotic and acyanotic CHD cases, implying that interventions should be implemented in line with CHD phenotypes and severity.

It is worth noting that pulmonary hypertension was the predictor of all three growth parameters, in keeping with a few studies (19, 26, 42) that advocate for heightened attention towards CHD cases with pulmonary hypertension. Despite this, pneumonia was associated with growth insufficiency. In contrast to our result, Batte et al. illustrated preoperative factors such as the history of pneumonia was not statistically related to undernutrition among children post-operation (14). One likely explanation is the corrective surgery triggered catch-up growth in patients (43). Poor feeding elevates the risk of infections, such as pneumonia, which conversely worsens children's nutritional condition, forming a vicious cycle. For one thing, children diagnosed with pneumonia often manifest high fever and tachypnoea, resulting in increased metabolic demands, while diminishing appetites, and causing hypoxia (44). For another, antibiotics, which served as treatments for infections, bring about changes in the diversity and richness of gastrointestinal microbiota, giving rise to gut dysfunction and lack of absorption (45).

It is also important to stress that artificial feeding was a contributary factor of undernutrition, compared with breastfeeding and mixed feeding. Davis et al. articulated the prominence of breastfeeding, including unparalleled properties of appropriate concentration of protein, antibodies, and digestive enzymes, in comparison with formula milk (46). In Haiti, children adhering to breastfeeding practices were less likely to be wasted (47). All the evidence highlights the significance of promoting breast-feeding to mothers with CHD children. Thus, we conducted a subgroup analysis for breastfed and non-breastfed children. Results demonstrated that associated factors of undernutrition differed strikingly between the two groups, indicating that suggestions should be specifically offered to children with diverse feeding patterns. Furthermore, a randomized-controlled trial indicated that CHD patients fed with human milk and energy supplementation/fortifier gained higher energy than counterparts only fed with breast milk (48). Considering the elevated energy consumption for children with cardiac defects, personalized feeding patterns should be further studied.

Our study carries the following limitations. Firstly, participants were selected from a single tertiary hospital, affecting the study population's representativeness, and thus, extrapolation may be restricted. Secondly, due to the inherent drawback of cross-sectional studies, we were unable to verify the causality of the associated factors and growing deficit in CHD children. Moreover, these data must be interpreted with caution because of recall bias and investigation bias, although we implemented measures including checking medical records and information in PHCH, and training investigators. Thirdly, our study did not assess potential influence factors such as dietary factors of patients and their nutritional status post-operation. Since 40.2% of cases aged before 6 months in our survey and infants at this age were mostly breastfed, it was difficult to investigate their dietary factors. Instead, we recorded their feeding patterns. Fourthly, our findings could be impacted by intricate interactions between the variables we examined, such as the potential impact of demographic traits on cardiac factors, possibly mediated by hospital presentations and management. Therefore, multi-centered prospective cohort study with larger sample size should be conducted in the future, so that prominent and modifiable factors contributing to undernourishment in children with CHD can be detected.

## 5 Conclusion

Our study reveals a high prevalence of undernutrition among preoperative CHD children in China. Various factors have been identified to be linked to undernourishment, including parents' occupational status, family income, number of children, maternal body mass index before pregnancy, gestational complications (e.g., placental abruption), and maternal exposure to noisy environment. Additionally, we have found correlations between undernutrition and preterm birth, low birth weight, pulmonary hypertension, pneumonia, and feeding patterns. It is worth noting that factors associated with the nutritional status of breastfed and non-breastfed, cyanotic and acyanotic, preterm and non-preterm children differ significantly, underscoring the need for tailored interventions. Confirming these associations and explore additional potential factors, such as patients' dietary factors, necessitates further research.

## Data Availability

The raw data supporting the conclusions of this article will be made available by the authors, without undue reservation.
