# Supplementary material for: Associated factors of undernutrition in children with congenital heart disease: a cross-sectional study
Source: Front Pediatr. 2024 Jan 29;12:1167460. doi: 10.3389/fped.2024.1167460 (PMC10859474; doi:10.3389/fped.2024.1167460)
Supplement: Supplementary file 1 [file Datasheet1.docx]

Supplementary Material

**Supplementary Table1. Definitions of exposures of interest**

| Exposures of interest | Definition |
| --- | --- |
| Frequency of foods intake | (1) Never intake: less than once per week. (2) Sometimes intake: once or twice per week. (3) Always intake: more than or equal to three times per week. |
| Smoking before pregnancy | Smoking for >6 months continuously or cumulatively, and smoking index >100 in the last 6 months (smoking index = number of cigarettes smoked per day*year). |
| Smoking during early stage of pregnancy | Average number of cigarettes actively smoked ≥1 per day during the first trimester of gestation. |
| Passive smoking | Being exposed to smoke for >15 minutes per day, ≥4 days per week, whether at home and in the workplace. |
| Drinking | Drinking any kind of alcohols more than once per day, consuming ≥2g of alcohol. |
| Drinking tea | Drinking any kind of tea more than or equal to once per day. |
| Drinking coffee | Drinking any kind of coffee more than or equal to once per day. |
| Using computers | (1) Seldom: <1h/d. (2) Sometimes: 1-4h/d. (3) Always: >4h/d. |
| Using cellphones | (1) Seldom: <1h/d. (2) Sometimes: 1-4h/d. (3) Always: >4h/d. |
| Experiencing negative events | Experiencing at least one traumatic life event, including separation of one’s spouse, divorce, bereaved of one's spouse, abortion or miscarriage, financial difficulties, the mother herself or her family member or her friend is seriously ill or injured, dismissal from the workplace, etc. |
| Folate use | Any use of folic acid in 3 months before pregnancy and/or during the first trimester pregnancy. |
| Feeding pattern | 1. Breast feeding: The child is fed exclusively with breast milk, or duration of time breastfeeding with the addition of dairy products (or complementary feeding) for <20% of child’s age. 2. Artificial feeding: 20% < duration of time breastfeeding with the addition of dairy products (or complementary feeding) <80% of the child’s age. 3. Mixed feeding: The child is fed with dairy products or complementary foods as the entire diet, or duration of time breastfeeding with the addition of dairy products (or complementary feeding) for ≥80% of child’s age. |
| Cyanosis | Mainly refers to central cyanosis, when the mixing of oxygenated and deoxygenated blood occurs, with deoxyHemoglobin (HHb) >50 g/L. |
| Premature birth | Children born <37 weeks of gestation. |
| Pneumonia | 1. Presence of respiratory symptoms, with or without fever. 2. Signs of moist rales and (or) increased respiratory rate. The criteria for increased respiratory rate observed for 1min when calm: <2 months of age: ≥60 breaths/min; 2 months of age~1 year ≥50 breaths/min; 1~5 years of age ≥40 breaths/min; >5 years of age ≥30 breaths/min. 3. Radiological evidence such as exudation and spots in the lungs on chest X-ray. |

**Supplementary Table 2. Parental demographic factors and patients’ related factors, associated with underweight, wasting and stunting in CHD children**

| variables | all | underweight | | | wasting | | | stunting | | |
| --- | --- | --- | --- | --- | --- | --- | --- | --- | --- | --- |
|  |  | n | prevalence(%) | p-value | n | prevalence(%) | p-value | n | prevalence(%) | p-value |
| **Maternal demographic factors** |  |  |  |  |  |  |  |  |  |  |
| Age at this pregnancy |  |  |  |  |  |  |  |  |  |  |
| <25 | 238 | 88 | 0.370 |  | 73 | 0.307 |  | 44 | 0.185 |  |
| 25~35 | 429 | 155 | 0.361 |  | 127 | 0.296 |  | 99 | 0.231 |  |
| ≥35 | 67 | 22 | 0.328 | 0.823 | 18 | 0.269 | 0.832 | 13 | 0.194 | 0.354 |
| Residence |  |  |  |  |  |  |  |  |  |  |
| Rural area | 481 | 182 | 0.378 |  | 150 | 0.312 |  | 112 | 0.233 |  |
| Urban area | 253 | 83 | 0.328 | 0.177 | 68 | 0.269 | 0.225 | 44 | 0.174 | 0.064 |
| Education level |  |  |  |  |  |  |  |  |  |  |
| Middle school and below | 357 | 129 | 0.361 |  | 109 | 0.305 |  | 88 | 0.246 |  |
| High school | 209 | 83 | 0.397 |  | 65 | 0.311 |  | 41 | 0.196 |  |
| College and above | 168 | 53 | 0.315 | 0.260 | 44 | 0.262 | 0.521 | 27 | 0.161 | 0.064 |
| Occupational status |  |  |  |  |  |  |  |  |  |  |
| Unemployed | 379 | 157 | 0.414 |  | 130 | 0.343 |  | 91 | 0.240 |  |
| Employed | 355 | 108 | 0.304 | 0.002^+^ | 88 | 0.248 | 0.005^+^ | 65 | 0.183 | 0.059 |
| Family annual income (RMB) |  |  |  |  |  |  |  |  |  |  |
| <50,000 | 401 | 167 | 0.416 |  | 135 | 0.337 |  | 95 | 0.237 |  |
| 50,000-100,000 | 240 | 68 | 0.283 |  | 64 | 0.267 |  | 43 | 0.179 |  |
| ≥100,000 | 93 | 30 | 0.323 | 0.002^+^ | 19 | 0.204 | 0.019^+^ | 18 | 0.194 | 0.200 |
| Number of children |  |  |  |  |  |  |  |  |  |  |
| ≤2 | 639 | 222 | 0.347 |  | 185 | 0.290 |  | 124 | 0.194 |  |
| >2 | 95 | 43 | 0.453 | 0.046^+^ | 33 | 0.347 | 0.250 | 32 | 0.337 | 0.002^+^ |
| Body mass index before this pregnancy |  |  |  |  |  |  |  |  |  |  |
| <18.5 (underweight) | 121 | 57 | 0.471 |  | 46 | 0.380 |  | 28 | 0.231 |  |
| 18.5-23.9 (healthy) | 476 | 158 | 0.332 |  | 133 | 0.279 |  | 91 | 0.191 |  |
| ≥24.0 (overweight/obese) | 137 | 50 | 0.365 | 0.017^+^ | 39 | 0.285 | 0.090 | 37 | 0.270 | 0.119 |
| **Paternal demographic factors** |  |  |  |  |  |  |  |  |  |  |
| Education level |  |  |  |  |  |  |  |  |  |  |
| High school and below | 570 | 219 | 0.384 |  | 182 | 0.319 |  | 127 | 0.223 |  |
| College and above | 164 | 46 | 0.280 | 0.015^+^ | 36 | 0.220 | 0.014^+^ | 29 | 0.177 | 0.205 |
| Occupational status |  |  |  |  |  |  |  |  |  |  |
| Unemployed | 47 | 27 | 0.574 |  | 22 | 0.468 |  | 9 | 0.191 |  |
| Employed | 687 | 238 | 0.346 | 0.002^+^ | 196 | 0.285 | 0.008^+^ | 147 | 0.214 | 0.715 |
| **Patients’ demographic factors** |  |  |  |  |  |  |  |  |  |  |
| Gender |  |  |  |  |  |  |  |  |  |  |
| Male | 406 | 141 | 0.347 |  | 122 | 0.300 |  | 90 | 0.222 |  |
| Female | 328 | 124 | 0.378 | 0.388 | 96 | 0.293 | 0.818 | 66 | 0.201 | 0.501 |
| **Birth-related factors** |  |  |  |  |  |  |  |  |  |  |
| Mode of delivery |  |  |  |  |  |  |  |  |  |  |
| Natural labor | 383 | 131 | 0.342 |  | 112 | 0.292 |  | 70 | 0.183 |  |
| Caesarean section | 351 | 134 | 0.382 | 0.263 | 106 | 0.302 | 0.777 | 86 | 0.245 | 0.039^+^ |
| Premature birth |  |  |  |  |  |  |  |  |  |  |
| No | 639 | 214 | 0.335 |  | 192 | 0.300 |  | 115 | 0.180 |  |
| Yes | 95 | 51 | 0.537 | 0.000^+^ | 26 | 0.274 | 0.594 | 41 | 0.432 | 0.000^+^ |
| Low birth weight |  |  |  |  |  |  |  |  |  |  |
| No | 636 | 202 | 0.318 |  | 184 | 0.289 |  | 108 | 0.170 |  |
| Yes | 98 | 63 | 0.643 | 0.000^+^ | 34 | 0.347 | 0.245 | 48 | 0.490 | 0.000^+^ |
| Multiple birth |  |  |  |  |  |  |  |  |  |  |
| No | 682 | 244 | 0.358 |  | 205 | 0.301 |  | 137 | 0.201 |  |
| Yes | 40 | 21 | 0.525 | 0.033^+^ | 11 | 0.275 | 0.731 | 19 | 0.475 | 0.000^+^ |
| **Cardiac-related factors** |  |  |  |  |  |  |  |  |  |  |
| Cyanosis |  |  |  |  |  |  |  |  |  |  |
| No | 647 | 223 | 0.345 |  | 186 | 0.287 |  | 132 | 0.204 |  |
| Yes | 87 | 42 | 0.483 | 0.012^+^ | 32 | 0.368 | 0.124 | 24 | 0.276 | 0.124 |
| Hemodynamics |  |  |  |  |  |  |  |  |  |  |
| Left to right shunt | 616 | 227 | 0.369 |  | 188 | 0.305 |  | 125 | 0.203 |  |
| Right to left shunt | 50 | 14 | 0.280 |  | 9 | 0.180 |  | 16 | 0.320 |  |
| No shunt | 68 | 24 | 0.353 | 0.451 | 21 | 0.309 | 0.172 | 15 | 0.221 | 0.148 |
| **Preoperative factors** |  |  |  |  |  |  |  |  |  |  |
| Pulmonary hypertension |  |  |  |  |  |  |  |  |  |  |
| No | 443 | 100 | 0.226 |  | 88 | 0.199 |  | 67 | 0.151 |  |
| Yes | 291 | 165 | 0.567 | 0.000^+^ | 130 | 0.447 | 0.000^+^ | 89 | 0.306 | 0.000^+^ |
| Pneumonia |  |  |  |  |  |  |  |  |  |  |
| No | 502 | 147 | 0.293 |  | 130 | 0.259 |  | 82 | 0.163 |  |
| Yes | 232 | 118 | 0.509 | 0.000^+^ | 88 | 0.379 | 0.001^+^ | 74 | 0.319 | 0.000^+^ |
| Feeding pattern |  |  |  |  |  |  |  |  |  |  |
| Breast feeding | 329 | 94 | 0.286 |  | 87 | 0.264 |  | 49 | 0.149 |  |
| Artificial feeding | 93 | 50 | 0.538 |  | 38 | 0.409 |  | 30 | 0.323 |  |
| Mixed feeding | 312 | 121 | 0.388 | 0.000^+^ | 93 | 0.298 | 0.027^+^ | 77 | 0.247 | 0.000^+^ |

^+^ statistically significant

^*^ correction for continuity

^#^ Fisher's exact probability method

**Supplementary Table 3. Association between history of adverse pregnancy outcomes, family history, and gestational complications during this pregnancy, and underweight, wasting and stunting in CHD children**

| variables | all | underweight | | | wasting | | | stunting | | |
| --- | --- | --- | --- | --- | --- | --- | --- | --- | --- | --- |
|  |  | n | prevalence(%) | p-value | n | prevalence(%) | p-value | n | prevalence(%) | p-value |
| History of adverse birth outcomes |  |  |  |  |  |  |  |  |  |  |
| No | 559 | 198 | 0.354 |  | 166 | 0.297 |  | 122 | 0.218 |  |
| Yes | 175 | 67 | 0.383 | 0.491 | 52 | 0.297 | 0.996 | 34 | 0.194 | 0.499 |
| History of spontaneous abortion |  |  |  |  |  |  |  |  |  |  |
| No | 633 | 232 | 0.367 |  | 192 | 0.303 |  | 136 | 0.215 |  |
| Yes | 101 | 33 | 0.327 | 0.440 | 26 | 0.257 | 0.349 | 20 | 0.198 | 0.701 |
| History of stillborn fetus |  |  |  |  |  |  |  |  |  |  |
| No | 709 | 256 | 0.361 |  | 212 | 0.299 |  | 153 | 0.216 |  |
| Yes | 25 | 9 | 0.360 | 0.991 | 6 | 0.240 | 0.526 | 3 | 0.120 | 0.250 |
| History of premature birth |  |  |  |  |  |  |  |  |  |  |
| No | 725 | 263 | 0.363 |  | 216 | 0.298 |  | 153 | 0.211 |  |
| Yes | 9 | 2 | 0.222 | 0.601* | 2 | 0.222 | 0.899* | 3 | 0.333 | 0.630* |
| History of low birth weight |  |  |  |  |  |  |  |  |  |  |
| No | 728 | 263 | 0.361 |  | 217 | 0.298 |  | 154 | 0.212 |  |
| Yes | 6 | 2 | 0.333 | 1.000* | 1 | 0.167 | 0.800* | 2 | 0.333 | 0.822* |
| History of intrauterine growth arrest |  |  |  |  |  |  |  |  |  |  |
| No | 707 | 249 | 0.352 |  | 204 | 0.289 |  | 152 | 0.215 |  |
| Yes | 27 | 16 | 0.593 | 0.011+ | 14 | 0.519 | 0.010+ | 4 | 0.148 | 0.405 |
| History of gestational complications |  |  |  |  |  |  |  |  |  |  |
| No | 638 | 231 | 0.362 |  | 186 | 0.292 |  | 138 | 0.216 |  |
| Yes | 96 | 34 | 0.354 | 0.881 | 32 | 0.333 | 0.403 | 18 | 0.188 | 0.520 |
| History of gestational diabetes mellitus |  |  |  |  |  |  |  |  |  |  |
| No | 730 | 262 | 0.359 |  | 215 | 0.295 |  | 155 | 0.212 |  |
| Yes | 4 | 3 | 0.750 | 0.270* | 3 | 0.750 | 0.150* | 1 | 0.250 | 1.000# |
| History of gestational hypertension |  |  |  |  |  |  |  |  |  |  |
| No | 720 | 259 | 0.360 |  | 212 | 0.294 |  | 151 | 0.210 |  |
| Yes | 14 | 6 | 0.429 | 0.595 | 6 | 0.429 | 0.428* | 5 | 0.357 | 0.315* |
| History of placenta previa |  |  |  |  |  |  |  |  |  |  |
| No | 727 | 263 | 0.362 |  | 216 | 0.297 |  | 155 | 0.213 |  |
| Yes | 7 | 2 | 0.286 | 0.983* | 2 | 0.286 | 1.000* | 1 | 0.143 | 1.000* |
| History of premature rupture of membrane |  |  |  |  |  |  |  |  |  |  |
| No | 729 | 262 | 0.359 |  | 215 | 0.295 |  | 155 | 0.213 |  |
| Yes | 5 | 3 | 0.600 | 0.516* | 3 | 0.600 | 0.319* | 1 | 0.200 | 1.000* |
| History of antepartum/postpartum hemorrhage |  |  |  |  |  |  |  |  |  |  |
| No | 705 | 255 | 0.362 |  | 206 | 0.292 |  | 152 | 0.216 |  |
| Yes | 29 | 10 | 0.345 | 0.853 | 12 | 0.414 | 0.160 | 4 | 0.138 | 0.316 |
| History of anemia during pregnancy |  |  |  |  |  |  |  |  |  |  |
| No | 678 | 246 | 0.363 |  | 204 | 0.301 |  | 146 | 0.215 |  |
| Yes | 56 | 19 | 0.339 | 0.724 | 14 | 0.250 | 0.423 | 10 | 0.179 | 0.518 |
| History of ectopic gestation |  |  |  |  |  |  |  |  |  |  |
| No | 712 | 257 | 0.361 |  | 212 | 0.298 |  | 152 | 0.213 |  |
| Yes | 22 | 8 | 0.364 | 0.979 | 6 | 0.273 | 0.800 | 4 | 0.182 | 0.926* |
| Family history of congenital malformation |  |  |  |  |  |  |  |  |  |  |
| No | 692 | 244 | 0.353 |  | 201 | 0.290 |  | 144 | 0.208 |  |
| Yes | 42 | 21 | 0.500 | 0.068# | 17 | 0.405 | 0.115 | 12 | 0.286 | 0.233 |
| Gestational complications during this pregnancy |  |  |  |  |  |  |  |  |  |  |
| No | 373 | 119 | 0.319 |  | 94 | 0.252 |  | 69 | 0.185 |  |
| Yes | 361 | 146 | 0.404 | 0.016+ | 124 | 0.343 | 0.007+ | 87 | 0.241 | 0.064 |
| Gestational diabetes mellitus during this pregnancy |  |  |  |  |  |  |  |  |  |  |
| No | 675 | 245 | 0.363 |  | 199 | 0.295 |  | 141 | 0.209 |  |
| Yes | 59 | 20 | 0.339 | 0.713 | 19 | 0.322 | 0.661 | 15 | 0.254 | 0.414 |
| Gestational hypertension during this pregnancy |  |  |  |  |  |  |  |  |  |  |
| No | 689 | 243 | 0.353 |  | 199 | 0.289 |  | 140 | 0.203 |  |
| Yes | 45 | 22 | 0.489 | 0.065 | 19 | 0.422 | 0.058 | 16 | 0.356 | 0.015+ |
| Placenta previa during this pregnancy |  |  |  |  |  |  |  |  |  |  |
| No | 694 | 246 | 0.354 |  | 202 | 0.291 |  | 150 | 0.216 |  |
| Yes | 40 | 19 | 0.475 | 0.123 | 16 | 0.400 | 0.143 | 6 | 0.150 | 0.320 |
| Placental abruption during this pregnancy |  |  |  |  |  |  |  |  |  |  |
| No | 729 | 261 | 0.358 |  | 218 | 0.299 |  | 152 | 0.209 |  |
| Yes | 5 | 4 | 0.800 | 0.113* | 0 | 0.000 | 0.333* | 4 | 0.800 | 0.008*+ |
| Premature rupture of membrane during this pregnancy |  |  |  |  |  |  |  |  |  |  |
| No | 715 | 254 | 0.355 |  | 211 | 0.295 |  | 146 | 0.204 |  |
| Yes | 19 | 11 | 0.579 | 0.045+ | 7 | 0.368 | 0.490 | 10 | 0.526 | 0.002*+ |
| Antepartum/postpartum hemorrhage during this pregnancy |  |  |  |  |  |  |  |  |  |  |
| No | 639 | 230 | 0.360 |  | 190 | 0.297 |  | 135 | 0.211 |  |
| Yes | 95 | 35 | 0.368 | 0.872 | 28 | 0.295 | 0.959 | 21 | 0.221 | 0.828 |
| Anemia during pregnancy during this pregnancy |  |  |  |  |  |  |  |  |  |  |
| No | 538 | 185 | 0.344 |  | 156 | 0.290 |  | 114 | 0.212 |  |
| Yes | 196 | 80 | 0.408 | 0.109 | 62 | 0.316 | 0.489 | 42 | 0.214 | 0.944 |

^+^ statistically significant

^*^ correction for continuity

^#^ Fisher's exact probability method

**Supplementary Table 4. Maternal periconceptional dietary factors, intake of folic acid, and exposure to environmental hazards associated with underweight, wasting and stunting in CHD children**

| variables | all | underweight | | | wasting | | | stunting | | |
| --- | --- | --- | --- | --- | --- | --- | --- | --- | --- | --- |
|  |  | n | prevalence(%) | p-value | n | prevalence(%) | p-value | n | prevalence(%) | p-value |
| **Dietary factors** |  |  |  |  |  |  |  |  |  |  |
| Eating pickles |  |  |  |  |  |  |  |  |  |  |
| Never | 378 | 145 | 0.384 |  | 121 | 0.320 |  | 81 | 0.214 |  |
| Sometimes | 322 | 107 | 0.332 |  | 89 | 0.276 |  | 66 | 0.205 |  |
| Always | 34 | 13 | 0.382 | 0.358 | 8 | 0.235 | 0.326 | 9 | 0.265 | 0.715 |
| Eating preserved eggs |  |  |  |  |  |  |  |  |  |  |
| Never | 589 | 214 | 0.363 |  | 182 | 0.309 |  | 122 | 0.207 |  |
| Sometimes | 136 | 46 | 0.338 |  | 32 | 0.235 |  | 32 | 0.235 |  |
| Always | 9 | 5 | 0.556 | 0.421^*^ | 4 | 0.444 | 0.145^*^ | 2 | 0.222 | 0.771^*^ |
| Eating salted eggs |  |  |  |  |  |  |  |  |  |  |
| Never | 605 | 218 | 0.360 |  | 185 | 0.306 |  | 130 | 0.215 |  |
| Sometimes | 123 | 45 | 0.366 |  | 32 | 0.260 |  | 26 | 0.211 |  |
| Always | 6 | 2 | 0.333 | 0.983^*^ | 1 | 0.167 | 0.452^*^ | 0 | 0.000 | 0.236^*^ |
| Eating smoked food |  |  |  |  |  |  |  |  |  |  |
| Never | 370 | 137 | 0.370 |  | 112 | 0.303 |  | 83 | 0.224 |  |
| Sometimes | 343 | 120 | 0.350 |  | 99 | 0.289 |  | 70 | 0.204 |  |
| Always | 21 | 8 | 0.381 | 0.836 | 7 | 0.333 | 0.858 | 3 | 0.143 | 0.571^*^ |
| Eating fried food |  |  |  |  |  |  |  |  |  |  |
| Never | 440 | 158 | 0.359 |  | 140 | 0.318 |  | 98 | 0.223 |  |
| Sometimes | 269 | 99 | 0.368 |  | 72 | 0.268 |  | 54 | 0.201 |  |
| Always | 25 | 8 | 0.320 | 0.884 | 6 | 0.240 | 0.295 | 4 | 0.160 | 0.635 |
| Eating dairy products |  |  |  |  |  |  |  |  |  |  |
| Never | 215 | 79 | 0.367 |  | 70 | 0.326 |  | 45 | 0.209 |  |
| Sometimes | 243 | 84 | 0.346 |  | 60 | 0.247 |  | 45 | 0.185 |  |
| Always | 276 | 102 | 0.370 | 0.830 | 88 | 0.319 | 0.111 | 66 | 0.239 | 0.322 |
| Eating eggs |  |  |  |  |  |  |  |  |  |  |
| Never | 56 | 27 | 0.482 |  | 19 | 0.339 |  | 12 | 0.214 |  |
| Sometimes | 207 | 75 | 0.362 |  | 68 | 0.329 |  | 44 | 0.213 |  |
| Always | 471 | 163 | 0.346 | 0.134 | 131 | 0.278 | 0.322 | 100 | 0.212 | 0.999 |
| Eating vegetables |  |  |  |  |  |  |  |  |  |  |
| Never | 5 | 2 | 0.400 |  | 0 | 0.000 |  | 2 | 0.400 |  |
| Sometimes | 30 | 10 | 0.333 |  | 8 | 0.267 |  | 6 | 0.200 |  |
| Always | 699 | 253 | 0.362 | 0.934^*^ | 210 | 0.300 | 0.157^*^ | 148 | 0.212 | 0.628^*^ |
| Eating fruits |  |  |  |  |  |  |  |  |  |  |
| Never | 15 | 6 | 0.400 |  | 3 | 0.200 |  | 4 | 0.267 |  |
| Sometimes | 101 | 33 | 0.327 |  | 31 | 0.307 |  | 23 | 0.228 |  |
| Always | 618 | 226 | 0.366 | 0.715 | 184 | 0.298 | 0.678^*^ | 129 | 0.209 | 0.803^*^ |
| Eating beans |  |  |  |  |  |  |  |  |  |  |
| Never | 104 | 39 | 0.375 |  | 33 | 0.317 |  | 27 | 0.260 |  |
| Sometimes | 366 | 139 | 0.380 |  | 112 | 0.306 |  | 83 | 0.227 |  |
| Always | 264 | 87 | 0.330 | 0.411 | 73 | 0.277 | 0.645 | 46 | 0.174 | 0.127 |
| Eating meat |  |  |  |  |  |  |  |  |  |  |
| Never | 13 | 3 | 0.231 |  | 2 | 0.154 |  | 3 | 0.231 |  |
| Sometimes | 79 | 32 | 0.405 |  | 24 | 0.304 |  | 19 | 0.241 |  |
| Always | 642 | 230 | 0.358 | 0.427^*^ | 192 | 0.299 | 0.478^*^ | 134 | 0.209 | 0.803^*^ |
| Eating seafood |  |  |  |  |  |  |  |  |  |  |
| Never | 104 | 43 | 0.413 |  | 32 | 0.308 |  | 28 | 0.221 |  |
| Sometimes | 374 | 147 | 0.393 |  | 119 | 0.318 |  | 83 | 0.795 |  |
| Always | 256 | 75 | 0.293 | 0.018^+^ | 67 | 0.262 | 0.303 | 45 | 0.544 | 0.119 |
| **Intake of folic acid** |  |  |  |  |  |  |  |  |  |  |
| No | 80 | 31 | 0.388 |  | 24 | 0.300 |  | 18 | 0.225 |  |
| Yes | 654 | 234 | 0.358 | 0.602 | 194 | 0.297 | 0.950 | 138 | 0.211 | 0.773 |
| **Exposure to environmental hazards** |  |  |  |  |  |  |  |  |  |  |
| Exposure to air pollution |  |  |  |  |  |  |  |  |  |  |
| No | 642 | 239 | 0.372 |  | 189 | 0.294 |  | 140 | 0.218 |  |
| Yes | 92 | 26 | 0.283 | 0.094 | 29 | 0.315 | 0.683 | 16 | 0.174 | 0.333 |
| Exposure to noisy environment |  |  |  |  |  |  |  |  |  |  |
| No | 575 | 208 | 0.362 |  | 158 | 0.275 |  | 127 | 0.221 |  |
| Yes | 159 | 57 | 0.358 | 0.940 | 60 | 0.377 | 0.012^+^ | 29 | 0.182 | 0.294 |
| Exposure to newly renovated houses |  |  |  |  |  |  |  |  |  |  |
| No | 657 | 232 | 0.353 |  | 195 | 0.297 |  | 136 | 0.207 |  |
| Yes | 77 | 33 | 0.429 | 0.192 | 23 | 0.299 | 0.972 | 20 | 0.260 | 0.285 |
| Exposure to radiation |  |  |  |  |  |  |  |  |  |  |
| No | 652 | 236 | 0.362 |  | 197 | 0.302 |  | 143 | 0.219 |  |
| Yes | 82 | 29 | 0.354 | 0.883 | 21 | 0.256 | 0.390 | 13 | 0.159 | 0.205 |

^+^ statistically significant

^*^ correction for continuity

**Supplementary Table 5. Parental life behaviors and habits associated with underweight, wasting and stunting in CHD children**

| variables | all | underweight | | | wasting | | | stunting | | |
| --- | --- | --- | --- | --- | --- | --- | --- | --- | --- | --- |
|  |  | n | prevalence(%) | p-value | n | prevalence(%) | p-value | n | prevalence(%) | p-value |
| **Maternal life habits before pregnancy** | | |  |  |  |  |  |  |  |  |
| Smoking before pregnancy |  |  |  |  |  |  |  |  |  |  |
| No | 713 | 256 | 0.359 |  | 212 | 0.297 |  | 152 | 0.213 |  |
| Yes | 21 | 9 | 0.429 | 0.513 | 6 | 0.286 | 0.909 | 4 | 0.190 | 1.000^*^ |
| Passive smoking before pregnancy |  |  |  |  |  |  |  |  |  |  |
| No | 359 | 127 | 0.354 |  | 111 | 0.309 |  | 68 | 0.189 |  |
| Yes | 375 | 138 | 0.368 | 0.688 | 107 | 0.285 | 0.479 | 88 | 0.235 | 0.134 |
| Drinking before pregnancy |  |  |  |  |  |  |  |  |  |  |
| No | 691 | 245 | 0.355 |  | 205 | 0.297 |  | 150 | 0.217 |  |
| Yes | 43 | 20 | 0.465 | 0.143 | 13 | 0.302 | 0.937 | 6 | 0.140 | 0.228 |
| Drinking tea before pregnancy |  |  |  |  |  |  |  |  |  |  |
| No | 670 | 239 | 0.357 |  | 199 | 0.297 |  | 143 | 0.213 |  |
| Yes | 64 | 26 | 0.406 | 0.431 | 19 | 0.297 | 0.998 | 13 | 0.203 | 0.847 |
| Drinking coffee before pregnancy |  |  |  |  |  |  |  |  |  |  |
| No | 714 | 261 | 0.366 |  | 214 | 0.300 |  | 153 | 0.214 |  |
| Yes | 20 | 4 | 0.200 | 0.128 | 4 | 0.200 | 0.336 | 3 | 0.150 | 0.677^*^ |
| **Maternal life habits during early stage of pregnancy** | | | | |  |  |  |  |  |  |
| Smoking during early stage of pregnancy |  |  |  |  |  |  |  |  |  |  |
| No | 725 | 260 | 0.359 |  | 216 | 0.298 |  | 155 | 0.214 |  |
| Yes | 9 | 5 | 0.556 | 0.382^*^ | 2 | 0.222 | 0.899^*^ | 1 | 0.111 | 0.735^*^ |
| Passive smoking during early stage of pregnancy |  |  |  |  |  |  |  |  |  |  |
| No | 448 | 157 | 0.350 |  | 137 | 0.306 |  | 92 | 0.205 |  |
| Yes | 286 | 108 | 0.378 | 0.455 | 81 | 0.283 | 0.514 | 64 | 0.224 | 0.552 |
| Drinking during early stage of pregnancy |  |  |  |  |  |  |  |  |  |  |
| No | 726 | 262 | 0.361 |  | 214 | 0.295 |  | 154 | 0.212 |  |
| Yes | 8 | 3 | 0.375 | 1.000^*^ | 4 | 0.500 | 0.382^*^ | 2 | 0.250 | 1.000^*^ |
| Drinking tea during early stage of pregnancy |  |  |  |  |  |  |  |  |  |  |
| No | 701 | 257 | 0.367 |  | 213 | 0.304 |  | 151 | 0.215 |  |
| Yes | 33 | 8 | 0.242 | 0.147 | 5 | 0.152 | 0.061 | 5 | 0.152 | 0.381 |
| Drinking coffee during early stage of pregnancy |  |  |  |  |  |  |  |  |  |  |
| No | 732 | 265 | 0.362 |  | 218 | 0.298 |  | 156 | 0.213 |  |
| Yes | 2 | 0 | 0.000 | 0.538^#^ | 0 | 0.000 | 1.000^#^ | 0 | 0.000 | 1.000^#^ |
| **Maternal periconceptional life behaviors** | | | |  |  |  |  |  |  |  |
| Using computers before pregnancy |  |  |  |  |  |  |  |  |  |  |
| Seldom | 528 | 192 | 0.364 |  | 161 | 0.305 |  | 118 | 0.223 |  |
| Sometimes | 91 | 32 | 0.352 |  | 24 | 0.264 |  | 20 | 0.220 |  |
| Always | 115 | 41 | 0.357 | 0.970 | 33 | 0.287 | 0.706 | 18 | 0.157 | 0.278 |
| Using cellphones before pregnancy |  |  |  |  |  |  |  |  |  |  |
| Seldom | 45 | 15 | 0.333 |  | 9 | 0.200 |  | 11 | 0.244 |  |
| Sometimes | 499 | 177 | 0.355 |  | 157 | 0.315 |  | 97 | 0.194 |  |
| Always | 190 | 73 | 0.384 | 0.712 | 52 | 0.274 | 0.195 | 48 | 0.253 | 0.214 |
| Using computers during early stage of pregnancy |  |  |  |  |  |  |  |  |  |  |
| Seldom | 610 | 215 | 0.352 |  | 179 | 0.293 |  | 130 | 0.213 |  |
| Sometimes | 56 | 20 | 0.357 |  | 13 | 0.232 |  | 12 | 0.214 |  |
| Always | 68 | 30 | 0.441 | 0.351 | 26 | 0.382 | 0.171 | 14 | 0.206 | 0.990 |
| Using cellphones during early stage of pregnancy |  |  |  |  |  |  |  |  |  |  |
| Seldom | 116 | 43 | 0.371 |  | 27 | 0.233 |  | 28 | 0.241 |  |
| Sometimes | 491 | 171 | 0.348 |  | 157 | 0.320 |  | 98 | 0.200 |  |
| Always | 127 | 51 | 0.402 | 0.522 | 34 | 0.268 | 0.133 | 30 | 0.236 | 0.474 |
| **Maternal exposure to stressful life events** | | | |  |  |  |  |  |  |  |
| Experiencing negative events half year before this pregnancy |  |  |  |  |  |  |  |  |  |  |
| No | 676 | 248 | 0.367 |  | 207 | 0.306 |  | 144 | 0.213 |  |
| Yes | 58 | 17 | 0.293 | 0.262 | 11 | 0.190 | 0.062 | 12 | 0.207 | 0.913 |
| Experiencing negative events during early stage of pregnancy |  |  |  |  |  |  |  |  |  |  |
| No | 643 | 232 | 0.361 |  | 195 | 0.303 |  | 136 | 0.212 |  |
| Yes | 91 | 33 | 0.363 | 0.973 | 23 | 0.253 | 0.324 | 20 | 0.220 | 0.857 |
| **Paternal lifestyle behaviors** |  |  |  |  |  |  |  |  |  |  |
| Smoking |  |  |  |  |  |  |  |  |  |  |
| No | 243 | 83 | 0.342 |  | 69 | 0.284 |  | 51 | 0.210 |  |
| Yes | 491 | 182 | 0.371 | 0.440 | 149 | 0.303 | 0.586 | 105 | 0.214 | 0.901 |
| Drinking |  |  |  |  |  |  |  |  |  |  |
| No | 408 | 144 | 0.353 |  | 116 | 0.284 |  | 83 | 0.203 |  |
| Yes | 326 | 121 | 0.371 | 0.610 | 102 | 0.313 | 0.400 | 73 | 0.224 | 0.500 |
| Drinking tea |  |  |  |  |  |  |  |  |  |  |
| No | 527 | 189 | 0.359 |  | 152 | 0.288 |  | 110 | 0.209 |  |
| Yes | 207 | 76 | 0.367 | 0.829 | 66 | 0.319 | 0.417 | 46 | 0.222 | 0.688 |
| Drinking coffee |  |  |  |  |  |  |  |  |  |  |
| No | 720 | 260 | 0.361 |  | 212 | 0.294 |  | 154 | 0.214 |  |
| Yes | 14 | 5 | 0.357 | 0.976 | 6 | 0.429 | 0.418^*^ | 2 | 0.143 | 0.754^*^ |
| Using computers |  |  |  |  |  |  |  |  |  |  |
| Seldom | 499 | 183 | 0.367 |  | 148 | 0.297 |  | 108 | 0.216 |  |
| Sometimes | 149 | 53 | 0.356 |  | 45 | 0.302 |  | 30 | 0.201 |  |
| Always | 86 | 29 | 0.337 | 0.861 | 25 | 0.291 | 0.983 | 18 | 0.209 | 0.922 |
| Using cellphones |  |  |  |  |  |  |  |  |  |  |
| Seldom | 42 | 11 | 0.262 |  | 11 | 0.262 |  | 7 | 0.167 |  |
| Sometimes | 492 | 177 | 0.360 |  | 138 | 0.280 |  | 106 | 0.215 |  |
| Always | 200 | 77 | 0.385 | 0.318 | 69 | 0.345 | 0.213 | 43 | 0.215 | 0.756 |

^+^ statistically significant

^*^ correction for continuity

^#^ Fisher's exact probability method
