# Supplementary figures and images for: Associated factors of undernutrition in children with congenital heart disease: a cross-sectional study
Source: Front Pediatr. 2024 Jan 29;12:1167460. doi: 10.3389/fped.2024.1167460 (PMC10859474; doi:10.3389/fped.2024.1167460)

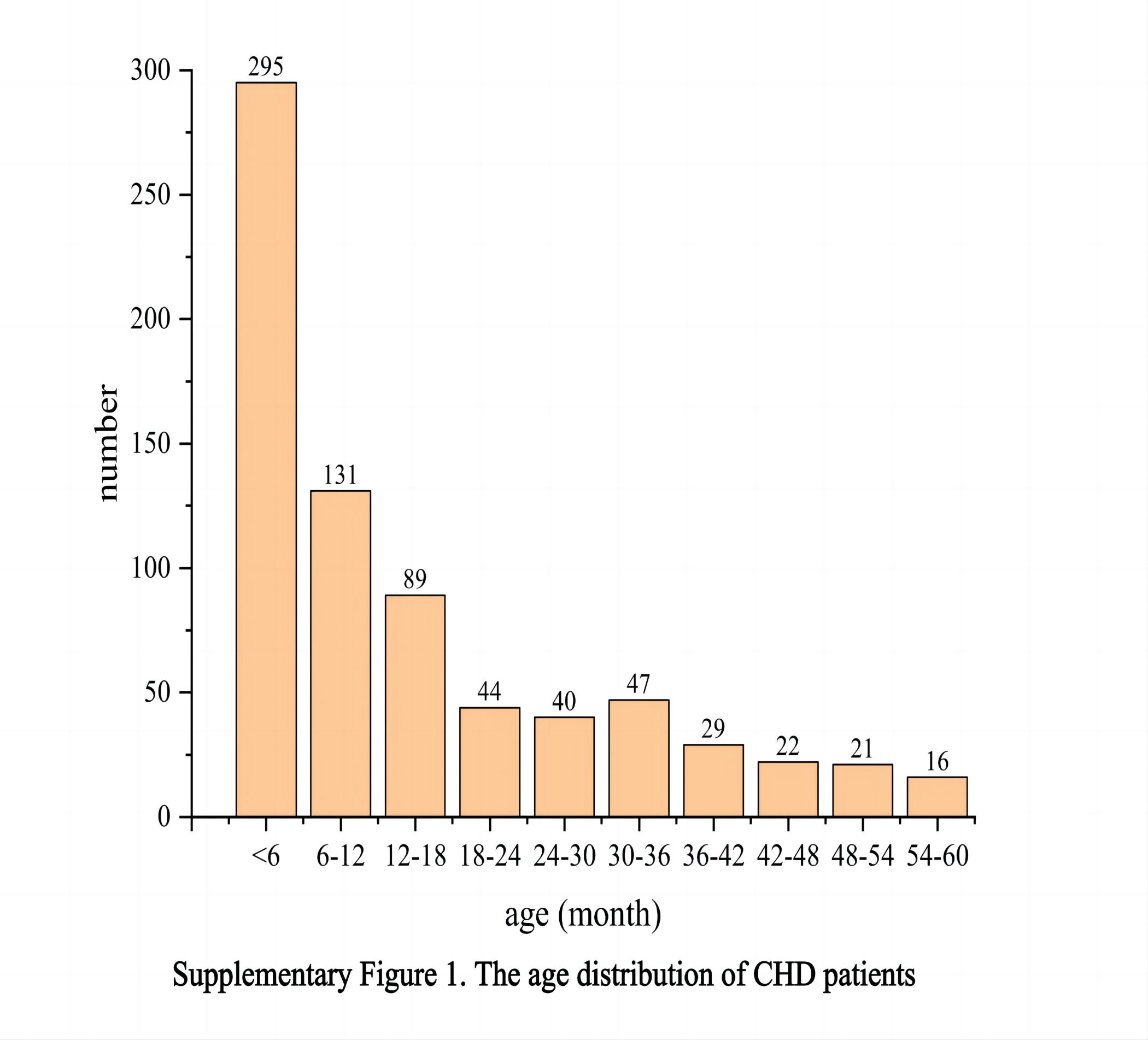

Supplement: Supplementary file 2 [file Image1.jpeg]
